# Transfer Investigations of Lipophilic Drugs from Lipid Nanoemulsions to Lipophilic Acceptors: Contributing Effects of Cholesteryl Esters and Albumin as Acceptor Structures

**DOI:** 10.3390/ph14090865

**Published:** 2021-08-28

**Authors:** Sabrina Knoke, Heike Bunjes

**Affiliations:** 1 Technische Universität Braunschweig, Institut für Pharmazeutische Technologie und Biopharmazie, Mendelssohnstraße 1, 38106 Braunschweig, Germany; sabrina.knoke@tu-braunschweig.de; 2 Technische Universität Braunschweig, Zentrum für Pharmaverfahrenstechnik (PVZ), Franz-Liszt-Straße 35a, 38106 Braunschweig, Germany

**Keywords:** drug transfer, in vitro release, colloidal drug carriers, lipid nanoparticles, hydrogel beads, cholesteryl nonanoate, bovine serum albumin

## Abstract

When studying the release of poorly water-soluble drugs from colloidal drug delivery systems designed for intravenous administration, the release media should preferentially contain lipophilic components that represent the physiological acceptors present in vivo. In this study, the effect of different acceptor structures was investigated by comparing the transfer of fenofibrate, retinyl acetate, and orlistat from trimyristin nanoemulsion droplets into lipid-containing hydrogel particles, as well as to bovine serum albumin (BSA). A nanodispersion based on trimyristin and cholesteryl nonanoate was incorporated into the hydrogel particles (mean diameter ~40 µm) in order to mimic the composition of lipoproteins. The course of transfer observed utilizing the lipid-containing hydrogel particles as an acceptor was in relation to the lipophilicity of the drugs: the higher the logP value, the slower the transfer. There was no detectable amount of the drugs transferred to BSA in liquid solution, demonstrating clearly that albumin alone does not contribute substantially as acceptor for the lipophilic drugs under investigation in this study. In contrast, cholesteryl nonanoate contributes to a much greater extent. However, in all cases, the partition equilibrium of the drugs under investigation was in favor of the trimyristin emulsion droplets.

## 1. Introduction

To overcome issues arising from the poor water solubility of many newly discovered drugs, lipid-based colloidal dispersions are under investigation as a promising formulation approach for the parenteral administration of these substances [1]. Such dispersions may, e.g., be liposomes, nanoemulsions, or may contain solid or liquid crystalline nanoparticles [2,3,4]. Information on the release behavior (or drug retention properties, respectively) of the lipid carrier particles is crucial for quality control, as well as to predict in vivo behavior. Since there is no officially approved test, efforts are being made to design appropriate setups for release testing of nanoparticulate drug carriers [5].

Challenges to face are, for example, related to the small size of the carrier particles. Only drugs with special properties, such as fluorescence, acidic/basic moieties, or electrochemically active groups, enable the detection of released drug with analytical methods that do not interfere with the dispersed phase particles [6,7,8]. Hence, many methods described in the literature require a separation step, such as filtration or centrifugation, in order to perform quantitative analysis of the released drug [9,10]. Other approaches are based on membrane barrier techniques [11,12,13] or continuous flow setups [14,15]. Depending on the method, the drug release behavior may be affected by the experimental conditions, e.g., due to high shear stress or long high-speed circulation times, insufficient time resolution, fluctuations in flow rates, or filter clogging [12,16,17,18,19].

For dosage forms that are designed to deliver lipophilic drugs via the intravenous (i.v.) route, release testing should be performed in an appropriate medium that reflects the physiological environment. Lipophilic compounds display poor aqueous solubility, and their distribution into mainly aqueous release media, for instance, simple buffer solutions, is limited. As an example, (lipo)proteins or cell compartments represent lipophilic acceptors in the blood that may be available for drug binding. Studies addressing this issue, e.g., investigated the transfer of lipophilic fluorescent dyes or temoporfin as model drugs into the oily droplets of o/w emulsions as acceptor using a flow cytometric approach [20], or focused on liposomes as acceptor, applying asymmetrical flow field-flow fractionation [21].

As an even closer approach to physiological conditions, Roese and Bunjes investigated drug transfer into porcine serum and blood using a method based on differential scanning calorimetry (DSC) for detection [22]. This method circumvents the necessity of separating the donor from the acceptor compartment, but may be limited in applicability to supercooled trimyristin donor nanoparticles and similar systems.

The highest proportion of proteins in human plasma can be attributed to albumin (~55%) [23]. Upon entering the bloodstream after parenteral administration, many drugs do not only associate with albumin, but also with lipoproteins [24]. Studies that analyzed the transfer properties of temoporfin from liposomes to some of the individual lipoprotein fractions and albumin in human plasma found significant differences regarding the distribution profiles of the drug [25,26]. After i.v. administration of liposomal amphotericin B, a large fraction of the drug was transferred to high density lipoproteins (HDL) [27]. For α-tocopherol, in contrast, low density lipoproteins (LDL) seem to be the predominant transport vehicle, as this substance was found to considerably associate with LDL after incubation in human plasma [28].

In order to predict the release performance in vivo, it is most desirable to investigate drug transfer into the original media relevant for administration. Unfortunately, the small size of the carrier particles on the one hand, as well as the complexity of the physiological environment present in vivo on the other hand, entails complications. During in vitro method development, it may thus be preferable to replace the very complex physiological media with simple and robust in vitro media that only contain the ingredients essential for the drug release process.

In a recent study, the transfer of lipophilic drugs from drug-loaded trimyristin emulsions was investigated using an unloaded trimyristin nanoemulsion incorporated into small calcium alginate hydrogel microbeads as a lipophilic acceptor [29]. This setup combined the advantages of small acceptor particles (with a large corresponding interfacial area) with a simple, filtration-based separation procedure from the donor particles. However, trimyristin emulsion droplets alone may not be sufficiently representative as components of “model-blood”, since other lipophilic substances, such as albumin and lipoproteins, might also have an impact on the drug distribution process. For example, LDL, which represent a large proportion of the plasma lipoprotein fraction, consist, for the most part, of cholesteryl esters [30,31].

To achieve an even closer approximation to the lipophilic acceptors in the blood, the trimyristin emulsion employed in the previous study was supplemented in the present study by the additional incorporation of a cholesteryl nonanoate dispersion into the hydrogel particles. Cholesteryl nonanoate was chosen as model cholesteryl ester as it forms nanodispersions in which it remains physically stable in a supercooled liquid crystalline state over a long period of time [3]. As another advantage, the saturated fatty acid chain of the cholesteryl nonanoate molecule is more resistant to chemical degradation, such as oxidation, in comparison to unsaturated derivates. It was an aim of this study to comprehensively characterize the trimyristin and cholesteryl nonanoate-containing hydrogel particles in order to perform transfer studies in a more advanced transfer medium. Using this approach, the contribution of the “model lipoprotein” acceptor hydrogel particles to the transfer of fenofibrate, retinyl acetate, and orlistat was investigated from trimyristin donor emulsions. Additionally, the transfer performance of these drugs was investigated from the same donor emulsions into albumin solution as acceptor by applying the DSC method for drug detection [22].

## 2. Results and Discussion

### 2.1. Characteristics of Donor and Acceptor Particles

#### 2.1.1. Particle Sizes

According to photon correlation spectroscopy (PCS) measurements, all intensity-weighted mean diameters (z-Averages) of the loaded and unloaded trimyristin (TM) nanoemulsions were between 113 and 126 nm, with polydispersity indices (PdIs) ≤0.10, indicating a monomodal size distribution. The z-Average diameters of the cholesteryl nonanoate (CN) dispersions and the mixed trimyristin–cholesteryl nonanoate (CNTM) dispersions were about 130 nm, and the PdIs were <0.11 (Table 1).

**Figure 1:**
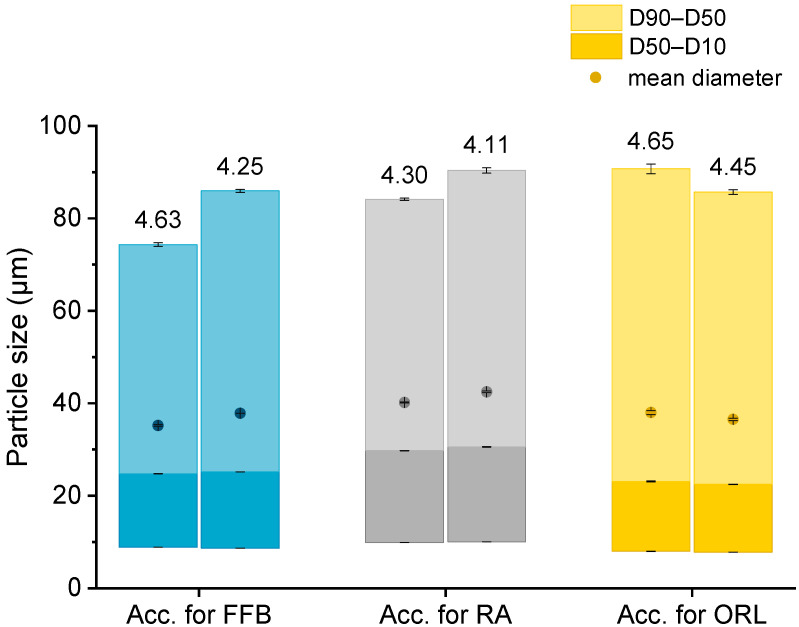
Particle size (represented as bars, all values *n* = 3 measurements ± SD) and lipid concentration (%) as determined by DSC (respective values above bars) of the different lipid-containing hydrogel bead dispersions utilized as acceptors (Acc.) in the respective transfer experiments. Further abbreviations: FFB—fenofibrate, RA—retinyl acetate, ORL—orlistat.

The particle sizes in each batch of the hydrogel particles containing the CNTM dispersion were determined via laser diffraction. In order to compare the drug transfer results obtained from this study with the results obtained from a previous study, hydrogel particles with a mean diameter between 35 and 42 µm were produced and utilized in transfer experiments (Figure 1). The D10 diameter was not below 8 µm so that the acceptor gel particles could be separated from the nanosized donor via filtration. The particle diameters, as well as the particle size distributions, were reproducible and very similar to those of the particles obtained in an earlier study [29].

#### 2.1.2. Drug Load of Donor Emulsions

Drug loading into the nanoemulsions was performed by dissolving the drug in the melted trimyristin prior to homogenization. All drugs were loaded in a concentration of ~3% in relation to the trimyristin matrix (based on lipid determination via DSC), leading to the final concentrations as shown in Table 1.

Please note that the given concentration of orlistat is the weighed-in amount, since it was not possible to determine orlistat with UV spectroscopy. This does not affect the accuracy of the calculated amount of transferred orlistat, as explained in Section 3.9. None of the drugs displayed significant adsorption on the PES filter membrane used during the transfer experiments, since recovery after filtration was close to 100% for all drugs (data not shown).

#### 2.1.3. Determination of Lipid Content

Knowledge of the lipid content in the nanodispersions, as well as in the lipid-containing hydrogel bead dispersions, was of essential importance for the adjustment of the lipid–mass ratio between donor and acceptor in the transfer studies. Differential scanning calorimetry (DSC) measurements were employed to evaluate the trimyristin content in the hydrogel particle dispersions, loaded and unloaded trimyristin-containing dispersions, whereas high performance liquid chromatography (HPLC) measurements were performed for the same unloaded emulsions, as well as the cholesteryl nonanoate dispersions. This procedure enabled a precise and verified lipid determination in the hydrogel bead dispersions and was necessary for two reasons. First, dispersed triglycerides are prone to forming different polymorphs that exhibit different crystallization enthalpies that may falsify the lipid quantification via DSC [32]. Second, it was not possible to completely dissolve the hydrogel beads in an appropriate solvent, such as tetrahydrofurane or acetonitrile, to extract the lipid in order to perform HPLC measurements. Thus, the lipid content in the acceptor dispersions had to be evaluated based on the DSC measurements and was calculated by multiplying the evaluated trimyristin content by 10, provided that the amount of cholesteryl nonanoate and trimyristin was accurately adjusted to 9 + 1. Comparing these two methods, the lipid determination of the TM and CNTM dispersions revealed very similar results in both cases (Table 1). Thus, applying the DSC method for the determination of the overall lipid content in the hydrogel bead dispersions seemed appropriate (please refer to the Supplementary Material for further detail). In general, the lipid content of the hydrogel bead dispersions was adjusted to ~4.4% by adding water (corresponding to approximately 44 mg/mL). A certain fraction of water surrounding the hydrogel particles was required to ensure thorough mixing of the donor and acceptor on the one hand, and to be able to draw a sufficient volume of sample out of the transfer vial for drug quantification on the other hand [29,33]. The lipid concentration of the hydrogel particle dispersions used for the transfer studies, as determined via DSC, are indicated in Figure 1.

#### 2.1.4. Structure Investigations

**Figure 2:**
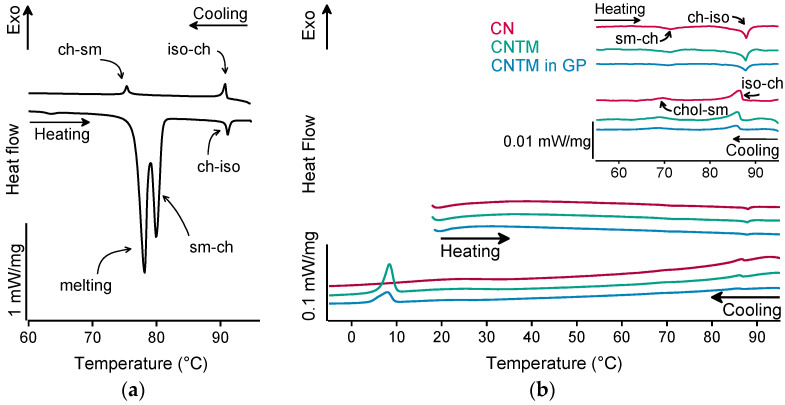
DSC curves of (**a**) cholesteryl nonanoate bulk material, (**b**) nanodispersions of pure cholesteryl nonanoate (CN), mixed cholesteryl nonanoate and trimyristin (CNTM), and the CNTM dispersion incorporated in hydrogel particles (CNTM in GP). The insert shows the transitions in the upper temperature range at a higher magnification. Further abbreviations: sm—smectic, ch—cholesteric, iso—isotropic melt. Scan rate: 5 K/min.

The different liquid crystalline phases of cholesteryl nonanoate can clearly be characterized by the combination of, e.g., thermal analysis and small-angle X-ray scattering (SAXS). As demonstrated in Figure 2a for the bulk material, the liquid crystalline phase transitions of cholesteryl nonanoate cause very small but distinct signals. Upon heating using DSC, the crystalline bulk material melted into the smectic mesophase and transformed immediately into the cholesteric mesophase at around 80 °C (Figure 2a) [3]. Upon further heating, the isotropic melt was formed at ~92 °C. Whilst cooling, the isotropic melt transformed back into the cholesteric phase which was present until transition into the smectic mesophase at around 76 °C. During heating of the dispersions (also inside the hydrogel beads), no melting peak was observed, demonstrating that all lipids were in a liquid (TM) or liquid crystalline (CN) state after production via hot melt homogenization (Figure 2b). The minor endothermic event occurring in all samples upon heating at about 72 °C corresponds to the transformation of CN from the smectic into the cholesteric mesophase. The main transition at about 87 °C is attributed to the melting of the cholesteric phase. The respective phase transitions upon cooling occurred in the same temperature range as upon heating. In comparison to the bulk material, a small temperature shift of the phase transitions was observed that is most likely related to the presence of the nanodispersed particles, as this was observed in another study as well [3]. However, the presence of trimyristin in the CNTM dispersion did not influence the formation of the liquid crystalline phases. No crystallization event was observed for CN within cooling to –10 °C, confirming the presence of the smectic mesophase of cholesteryl nonanoate. The exothermic event at <10 °C corresponds to the crystallization signal of the supercooled trimyristin droplets that was used for quantification (cf. Section 3.6).

**Figure 3:**
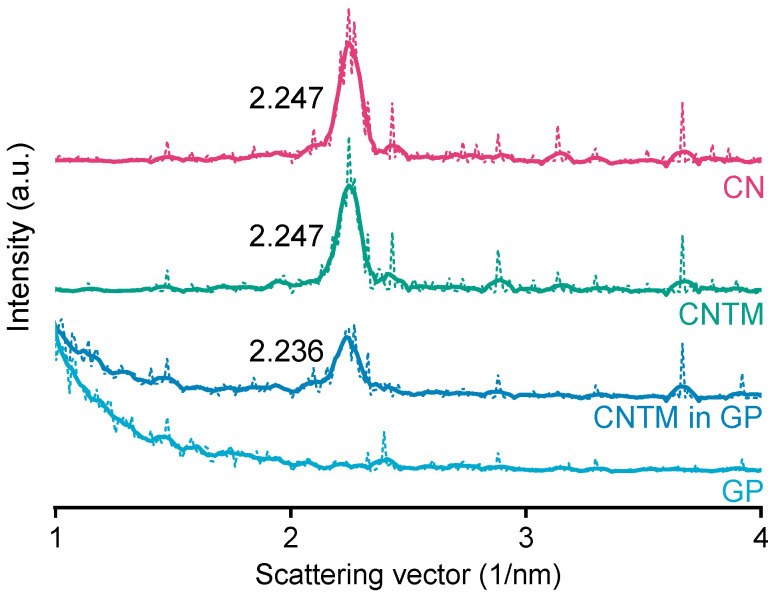
SAXS patterns of different nanodispersions and hydrogel beads at 20 °C. The values state the peak positions (q) upon which the calculation of the d-spacings was based. Abbreviations: CN—cholesteryl nonanoate, CNTM—cholesteryl nonanoate–trimyristin, GP—gel particle.

The smectic mesophase of cholesteryl nonanoate can be clearly identified by its very sharp and characteristic SAXS reflection [34]. The d-spacing of the CN dispersion was calculated to be 28.0 Å at about 20 °C (Figure 3), which is within the same range as literature data [3]. Neither the addition of trimyristin, nor encapsulation into the hydrogel matrix led to a prominent shift of the reflection (calculated to be 28.0 Å for the CNTM dispersion and 28.1 Å when incorporated into the hydrogel particles). These findings are in accordance with the phase transition events observed by DSC and confirm the presence of the smectic mesophase of CN in all dispersions, also, when incorporated in the hydrogel beads. The strong increase in scattering intensity at lower angles observed for the sample containing the CNTM dispersion in the hydrogel beads seemed to be caused by the hydrogel network, since the same phenomenon was observed by measuring the lipid-free hydrogel particles as control.

**Figure 4:**
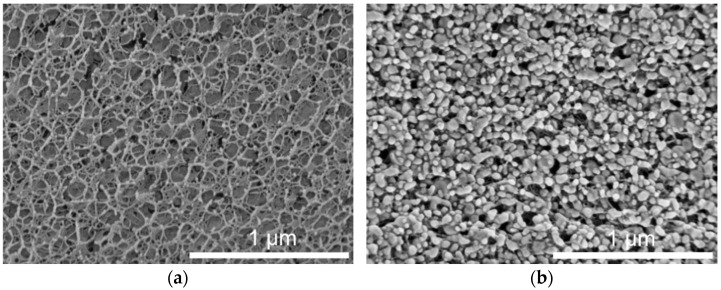
Cryo-SEM cross-sections of hydrogel particles. (**a**) Pure crosslinked alginate matrix. (**b**) Hydrogel particle containing mixed cholesteryl nonanoate–trimyristin dispersion.

Preserving the properties of the incorporated lipid nanoparticles was an important aim for the use of lipid-containing hydrogel microspheres as acceptor in the transfer experiments. During spraying upon hydrogel bead production, the lipid-containing alginate dispersion was exposed to high shear forces, which may have a negative effect on the integrity or state of the dispersed particles. Cryo-scanning electron microscopy (cryo-SEM) measurements were performed to depict the inner structure of the plain placebo and the lipid-containing hydrogel beads. The cryo-SEM images illustrate that the nanoparticles were associated with the hydrogel network and that their individuality seemed to be preserved in most cases (Figure 4). This is in accordance with the results obtained in earlier studies, in which the melting pattern of incorporated trimyristin particles was analyzed, and which indicated the presence of small nanoparticles inside the hydrogel beads [29].

The shape of the particles in the images implied that the incorporated lipids were no longer in their initial state (supercooled liquid in the case of trimyristin or smectic state in the case of cholesteryl nonanoate, as verified via DSC and SAXS), but seemed to be solidified due to the sample preparation procedure. In spite of that, the formation of the characteristic platelet-like shape of the crystalline lipid nanoparticles [32,35] seems to have been prevented, e.g., by the extremely rapid high-pressure freezing process or due to limited available space inside the hydrogel pores.

### 2.2. Investigation of Drug Transfer

#### 2.2.1. Transfer into CNTM-Containing Hydrogel Beads as Acceptor

**Figure 5:**
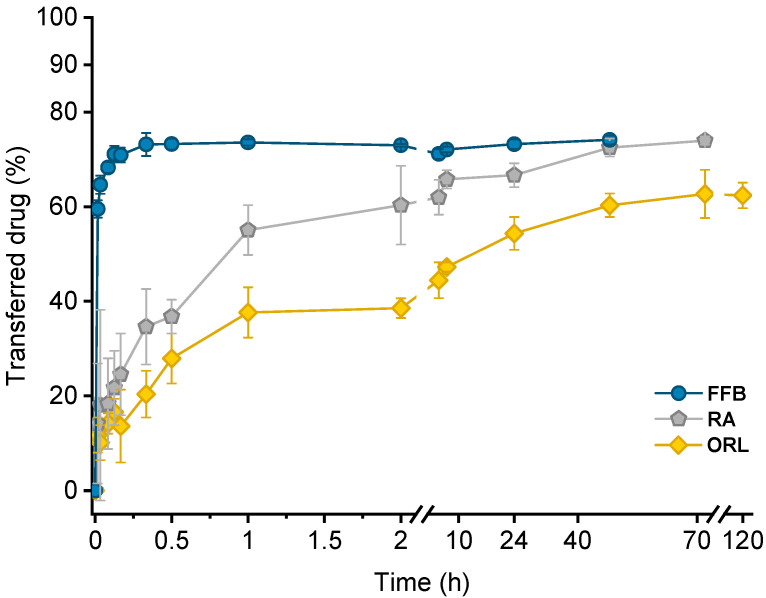
Drug transfer into CNTM-containing hydrogel beads. Each value represents mean ± SD (*n* = 3). Abbreviations: FFB—fenofibrate, RA—retinyl acetate, ORL—orlistat.

Drug transfer from trimyristin emulsions (donor; d) into CNTM-containing alginate microspheres (acceptor; a) was investigated for three different drugs loaded at a concentration of ~3% (drug related to matrix lipid). After mixing of donor and acceptor, fenofibrate (logP 4.86) transfer was completed within a few minutes, whereas retinyl acetate (logP 6.56) transferred more slowly and reached equilibrium at >40 h. Orlistat (logP 7.61) transfer seemed to be completed after about 70 h (Figure 5). The transfer course of all drugs was very similar to that obtained in earlier studies using trimyristin-nanodroplet-containing hydrogel microbeads as acceptor; however, the extent of drug transfer was distinctly smaller in the present case [29]. Assuming an equal distribution between the donor and acceptor lipids, a maximum fraction of 90% transferred drug would have been expected based on the adjusted lipid–mass ratio of 1 + 9 (d + a). In this case, however, the maximum fraction of transferred drug was ~74% for fenofibrate and retinyl acetate, whereas orlistat transferred to an extent of about 62%. The observed concentration equilibrium of the drugs was clearly shifted in favor of the donor. Thus, the affinity of all drugs seemed to be higher to trimyristin droplets instead of to the cholesteryl nonanoate particles. Bearing in mind that the hydrogel particles contained CN and TM in a mixture of 9 + 1, the contribution of the cholesteryl nonanoate as acceptor seemed very small in comparison to the trimyristin. Partition of the drugs can be assumed to be equal between the trimyristin of the donor emulsion and the trimyristin acceptor droplets that were incorporated in the hydrogel particles. In the case of fenofibrate and retinyl acetate, about 26% of the drugs were not transferred to the acceptor compartment but remained in the trimyristin donor emulsion. As a consequence, only approximately 50% of fenofibrate or retinyl acetate are presumably located in the cholesteryl nonanoate acceptor particles, considering that one tenth of the acceptor lipid is also composed of trimyristin. For orlistat, only ~62% of drug transfer to the acceptor particles was observed, corresponding to about 30% of the drug that is located in the cholesteryl nonanoate particles.

Reasons for this observation may be related to the lower lipophilicity of CN (CN eluted prior to TM from an RP column in the HPLC measurements). It may also be attributed to the presence of the liquid crystalline state. In the more ordered state of the liquid crystalline mesophase, the cholesteryl ester molecules are motionally restricted, which possibly made it more difficult for the drugs to associate with them [34]. Cholesteryl nonanoate was chosen as a model compound to mimic the cholesteryl ester fraction in lipoproteins present in the blood. At body temperature, LDL undergoes a phase transition, which is predominantly related to the phase transition of the cholesteryl esters into a more disordered, liquid-like state [30,36]. It might be conceivable that the drug transfer will be affected by the phase transition of LDL present in vivo. This remains to be investigated.

Lipophilicity, estimated based on the calculated logP values of the drugs, was a major factor determining the course of the drug transfer, which indicates that the transfer observed in these experiments is a partition driven process and that the characteristics of the drug (and not those of the carrier system) dominate the release performance. These findings are in accordance with the literature since drug release from lipid nanoemulsions has previously been reported to occur very rapidly [9,17,20,22]. A diffusion barrier due to the presence of the hydrogel matrix appeared not to be experimentally relevant for fenofibrate but seemed to be more critical for rather slowly transferring drugs, as already described in an earlier study in more detail [29].

#### 2.2.2. Bovine Serum Albumin (BSA) as Acceptor

In order to investigate albumin as acceptor for the lipophilic drugs under investigation, all drug-containing donor emulsions were incubated in BSA solution over 24 h. Directly after sampling at different time points, the samples were cooled from 25 °C to 0 °C via DSC, and the crystallization temperature of trimyristin (T_cryst._) was evaluated. Using this procedure, no filtration step was required, but control experiments were performed by incubating drug-free nanoemulsion in the BSA solution, as well as in pure PBS buffer.

**Figure 6:**
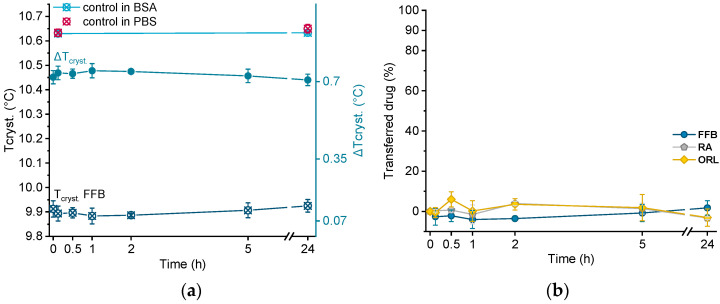
(**a**) Crystallization temperatures (T_cryst._) of unloaded trimyristin emulsion (control) and fenofibrate-containing emulsion (FFB), as well as the change of T_cryst._ (ΔT_cryst._) within 24 h. (**b**) Transferred percentage of fenofibrate (FFB), retinyl acetate (RA), and orlistat (ORL) to the BSA fraction.

Crystallization temperatures of control samples, as well as of a drug-loaded donor emulsion (exemplarily for fenofibrate), are plotted in Figure 6a. The presence of BSA solution did not influence the crystallization temperature of the unloaded trimyristin emulsion, which remained constant at about the same value as when incubated in pure PBS buffer (after 24 h, 10.63 ± 0.01 °C in BSA and 10.65 ± 0.02 °C in PBS, respectively). Consequently, obvious changes in T_cryst._ of the drug-loaded samples should only result from drug transfer from the nanoemulsion to BSA. Within the time frame of the experiment, the crystallization temperature of the FFB donor emulsion remained unchanged, but within the standard fluctuations of the measurement device. Consequently, the change of the crystallization temperature (ΔT_cryst._) of the fenofibrate-containing trimyristin emulsion in comparison to the unloaded nanoemulsion used as control remained very similar, indicating no transfer to BSA (Figure 6a,b).

Moreover, for retinyl acetate and orlistat no clear change in crystallization temperature was detected beyond the deviations of the measurement device, indicating that virtually no drug transferred to BSA (Figure 6b).

In previous studies, albumin was added to the aqueous release media as a solubilizing agent to enhance drug release from lipid nanocarriers. For example, Magenheim et al. and Levy and Benita investigated the release of miconazole and diazepam from triglyceride nanoemulsions applying sink conditions and found an increased amount of released drug in comparison to pure Hepes buffer [9,11].

In contrast, Reshetov et al. found no significant proportion of temoporfin bound to albumin in the presence of other lipophilic components [37]. Studies investigating the transfer of orlistat through oil–water interfaces revealed no effect on the transfer of orlistat by adding albumin, and found the partitioning of orlistat to be in favor of the oil phase, regardless of the composition of the aqueous phase [38]. These findings are in accordance with the results presented in this study.

In the case of fenofibrate, Pas et al. reported an increased solubility by a multiple in comparison to pure water. However, the absolute concentration did not exceed ~0.006 mg/mL [39]. The lack of transfer of fenofibrate to BSA in the present study is, thus, not particularly surprising, considering that the fenofibrate concentration applied in the present experiments was ~0.3 mg/mL. It might be conceivable that a very small amount of fenofibrate (and, consequently, retinyl acetate and orlistat) did transfer to BSA (up to their respective solubility limit), but was not detectable using the DSC method in this case. Dilution by many orders would seem to be required in this case to attain appropriate conditions that allow an adequate amount of drug to partition into the release medium.

In spite of that, it was clearly demonstrated that BSA, used here to mimic HSA, alone does not contribute substantially as an acceptor for the lipophilic drugs under investigation in this study. In contrast, cholesteryl nonanoate, utilized as a model compound to mimic the cholesteryl ester portion in lipoproteins, does contribute to a much greater extent. Yet, the partitioning of the drugs was in favor of the trimyristin in all cases. With regard to intravenous administration in humans, a significantly greater dilution, e.g., 1 + 1000, would be necessary to attain an even more realistic approach. For drugs under partition control, an increased transfer may be expected in vivo, if sufficient acceptor is provided. This remains to be investigated in further detail.

## 3. Materials and Methods

### 3.1. Materials

The triglyceride trimyristin (Dynasan^®^ 114) was donated by IOI Oleo, Witten, Germany, and the surfactant poloxamer 407 (Kolliphor^®^ P127) by BASF AG, Ludwigshafen, Germany. Sodium alginate (Manugel^®^ GMB) was a kind gift from FMC International, Wallingstown, Ireland. As estimated by the supplier, the molecular weight was ~124 kDa, the content of guluronic acid was 60–70%, and that of mannuronic acid was 30–40%. Cholesteryl nonanoate was purchased from TCI, Zwijndrecht, Belgium. Tetrahydrofuran (HPLC grade), acetonitrile (HPLC grade), bovine serum albumin (BSA, heat shock fraction, pH 7, ≥98%), and the drugs fenofibrate and retinyl acetate were obtained from Sigma-Aldrich, Steinheim, Germany. Orlistat was donated by Formosa Laboratories Inc., Taoyuan, Taiwan. Sodium azide, anhydrous glycerol, calcium chloride, acetonitrile (LC MS grade), and tetrahydrofuran (ultra LC MS grade) were obtained from Carl Roth, Karlsruhe, Germany. All materials were used as received. Water was purified by deionization and filtration (EASYpureTM LF, Barnstead, Dubuque, IA, USA) or was of bidistilled quality. The logP values of the drugs were obtained from DrugBank (calculated by ALOGPS).

### 3.2. Preparation of Donor and Acceptor Lipid Nanodispersions

The nanoemulsions consisted of 10% trimyristin (TM) as lipid phase, which was dispersed in an aqueous phase containing 5% poloxamer 407 as a stabilizer. The aqueous phase was isotonized with 2.25% anhydrous glycerol. Additionally, nanodispersions were prepared that contained 10% cholesteryl nonanoate (CN) as lipid phase. These dispersions were stabilized with 8% poloxamer 407. All lipid nanodispersions were preserved with 0.05% sodium azide. The concentrations are given related to the total weight of the dispersions (*w/w*).

The aqueous and lipid phases were preheated separately to 75 °C (TM nanoemulsions) or 95 °C (CN dispersions). After mixing, a pre-emulsion was formed using an Ultra-Turrax (T25 digital, IKA, Staufen, Germany) for four minutes at 11,000 rpm. Subsequently, the mixture was processed in the heat by high-pressure homogenization in 10 cycles at 700 bar (TM emulsions) or 900 bar (CN dispersions) using a Microfluidizer (M110-PS, interaction chamber type F12Y DIXC, Microfluidics, Newton, MA, USA).

After homogenization, all dispersions were filtered through a polyvinylidene fluoride (PVDF) filter with 0.45 μm pore size (Rotilabo^®^, Karlsruhe, Germany) and stored in glass vials at 20 °C. Under these conditions, TM remained in a liquid state due to supercooling [22], whereas the CN transformed into a liquid crystalline state [3]. The trimyristin nanoemulsion and the cholesteryl nonanoate dispersion were mixed in a ratio of 9 CN + 1 TM (CNTM dispersion; lipid ratio based on quantification via HPLC), and served as the acceptor system to be incorporated in alginate beads (cf. Section 3.4).

For the preparation of donor emulsions to be studied in transfer experiments, fenofibrate, retinyl acetate, or orlistat were dissolved in the melted trimyristin prior to emulsification. In order to exclude any possible influence of the drug loading on the transfer kinetics, all emulsions were loaded at the same concentration of ~3% related to trimyristin.

### 3.3. Lipid Quantification via High Performance Liquid Chromatography

A slight reduction in the lipid concentration may occur during dispersion production by dilution with process water remaining in the homogenization device. In order to achieve the lipid mixing ratio of 9 CN + 1 TM accurately, the lipid content of the unloaded dispersions was determined by HPLC after preparation. A Dionex UltiMate 3000 HPLC system (Thermo Fisher Scientific, Waltham, MA, USA) equipped with an LPG-3400SD pump, a WPS-3000TSL autosampler, and a Corona Veo Charged Aerosol detector was used to perform the analysis. The column (Thermo Fisher Scientific Hypersil Gold C18, 2.1 × 150 mm, 1.9 μm) was kept at 25 °C and the flow rate was set to 0.3 mL/min. The mobile phase consisted of acetonitrile/tetrahydrofuran 70/30 (*v/v*). Under these conditions, the retention time of both lipids was between 3 and 5 min, with cholesteryl nonanoate eluting from the column prior to trimyristin.

For sample preparation, dispersions were dissolved in tetrahydrofuran/acetonitrile 50/50 (*v/v*) and diluted to an appropriate detector response; 1 μL was injected and detected at a nebulizer temperature of 50 °C. Every sample was diluted twice and every dilution measured two times (*n* = 4). Lipid concentrations were calculated with the Chromeleon 7.2 software (Thermo Fisher Scientific, Waltham, MA, USA) using a calibration curve for trimyristin or cholesteryl nonanoate in different concentrations.

### 3.4. Preparation of Lipid-Containing Alginate Beads

Calcium alginate beads were produced with a spraying method as described earlier, with minor modifications [29]. The lipid nanodispersion that was incorporated into the hydrogel beads was composed of cholesteryl nonanoate and trimyristin (lipid mixing ratio 9 + 1 as determined by HPLC). The drug-free lipid nanodispersion was mixed with the same volume of bidistilled water (final volume approximately 20–25 mL each per batch) and 2% (*w/w*) sodium alginate was added to the dispersion. Under stirring at 200 rpm, the mixture was left to swell overnight. With the aid of a syringe pump (Fusion 200, Chemyx, Stafford, TX, USA), the resulting alginate-containing dispersion was fed (1 mL/min) into the two-fluid spray nozzle (diameter: 0.7 mm) of a BÜCHI Mini Spray Dryer B-191 (BÜCHI Labortechnik AG, Flawil, Switzerland) and sprayed under compressed air (650 L/h) into a continuously stirred 5% (*w/w*) CaCl_2_ solution (approximately 500 mL). The hydrogel particles were stored in the CaCl_2_ solution overnight to ensure thorough cross-linking. After hardening, excess CaCl_2_ was washed off with purified water via centrifugation (SIGMA^®^ 3–15, Sigma Laborzentrifugen GmbH, Osterode am Harz, Germany) three times at 3200 rpm. The microbeads were stored in water and used as acceptor particles. The resulting volume of each batch of dispersion was about 40–50 mL in total. The overall lipid concentration encapsulated in each batch of hydrogel bead dispersion was evaluated via DSC (cf. Section 3.6). Plain microbeads used for the cryo-SEM and SAXS analysis were produced in the same way but without lipid dispersion.

### 3.5. Particle Size Analysis

The particle size of the lipid nanodispersions was measured by PCS using a Zetasizer Nano ZS (Malvern Instruments, Worcestershire, UK). Prior to the measurement, samples were diluted with purified water to an appropriate scattering intensity (attenuator 5–7). After an equilibration time of 300 s, three consecutive measurements of 5 min each were performed at 25 °C using a laser wavelength of 633 nm at an angle of 173°. As an average of three runs, the z-Average and the PdI were calculated.

The particle sizes of the hydrogel beads were determined via laser diffraction (LD; Beckman Coulter LS 13 320, Beckman Coulter GmbH, Krefeld, Germany). The samples were diluted with water to an appropriate optical density in the measuring chamber. Three consecutive measurements of 90 s each were averaged and the volume distribution, mean particle size, and D10, D50, and D90 values were calculated using Fraunhofer approximation.

### 3.6. Differential Scanning Calorimetry

Differential scanning calorimetry (DSC) measurements were carried out using a DSC 1 calorimeter (Mettler Toledo, Gießen, Germany) equipped with an FRS 5+ sensor that was calibrated with indium. The calibration was checked by measuring indium before a series of measurements. About 20 mg of the samples were accurately weighed into 40 µL aluminum pans (Mettler Toledo, Gießen, Germany), which were hermetically sealed by cold welding. An empty pan was used as reference and all measurements were performed under nitrogen purge.

To examine the trimyristin concentration in the hydrogel bead dispersion, as well as in the drug-loaded and unloaded trimyristin-containing dispersions, samples were heated from 20 °C to 70 °C (20 K/min) and subsequently cooled to −5 °C with a scan rate of 10 K/min. The crystallization enthalpies from the cooling curves were evaluated and the trimyristin content was calculated using a calibration curve obtained from measuring different amounts of bulk trimyristin under the same conditions. The overall lipid content in mixed CNTM dispersions (composed of 9 CN + 1 TM), as well as the lipid amount in the hydrogel particle dispersions, was calculated by multiplying the determined trimyristin amount by 10.

The onset value of the crystallization signal of trimyristin was determined as an indicator for the crystallization temperature (T_cryst._). Samples from transfer experiments were cooled from 25 °C to 0 °C with a scan rate of 2.5 K/min. The changes in T_cryst._ of drug-loaded nanoemulsions were used to quantify the transferred amount of drug, as described in earlier studies; cf. Section 3.9.

In order to obtain information on the liquid crystalline structure, the cholesteryl no-nanoate dispersion and the CNTM-containing nanodispersion (also enclosed in the hydrogel beads) were heated from 15 °C to 100 °C (5 K/min), and subsequently cooled to −10 °C (5 K/min). The phase transitions indicated in the resulting curves were examined to characterize the structure of the incorporated lipid particles in comparison to those of unencapsulated counterparts.

If necessary, baseline correction was performed using OriginLab 2018.

### 3.7. Cryo-SEM

To evaluate the inner appearance of the placebo and the lipid-containing hydrogel beads, cryo-scanning electron microscopy (cryo-SEM) was performed using a Helios G4 CX DualBeam system and a Through the Lens Detector (FEI, Hillsboro, OR, USA). For sample preparation, the hydrogel microbeads were frozen in a high-pressure freezer (Leica EM ICE, Leica Microsystems GmbH, Wetzlar, Germany) using liquid nitrogen, subsequently fractured in the cryo-chamber at −150 °C, and sputtered with a 4 nm platinum layer in a high-vacuum coater (Leica EM ACE600, Leica Microsystems GmbH, Wetzlar, Germany). Imaging was performed at a voltage of 3 kV at different magnifications. The samples were kept under cryo conditions throughout the entire workflow.

### 3.8. X-ray Scattering

Small-angle X-ray scattering (SAXS) was performed to investigate the liquid crystalline structure of the cholesteryl-nonanoate-containing dispersions. The measurements were conducted with a SAXSess mc2 system (Anton Paar GmbH, Graz, Austria) using Cu Kα radiation (λ = 0.154) and a CCD detector (measurement range: q = 0 − 6 nm^−1^). The nanodispersions, as well as hydrogel beads (lipid-containing and placebo), were measured at room temperature in a 1 mm capillary, which was positioned in the beam path at a distance of 309 mm to the CCD detector.

Background and dark current subtraction, as well as desmearing, were performed using the SAXSquant software (Anton Paar GmbH, Graz, Austria). The raw data (dotted lines in Figure 3) were appropriately smoothed (solid lines; Figure 3), and the scattering vector q (nm^−1^) was determined using OriginLab 2018. The position of the reflections was used to calculate the layer spacing (d) according to Bragg’s law: d = 2π/q.

### 3.9. Investigation of Drug Transfer

#### 3.9.1. CNTM-Containing Hydrogel Beads as Acceptor

The procedure for drug transfer investigations using lipid-containing hydrogel beads as an acceptor was described earlier [29,33]. Briefly, drug-loaded nanoemulsions were mixed with the microsphere dispersion in 3 mL glass vials in a donor (d) to acceptor (a) lipid mass ratio of 1 + 9 (based on the results of lipid determination by DSC). This lipid ratio was chosen to ensure comparability of the present transfer results with those of a previous study [29]. The transfer started when the donor emulsion (~70 µL) was added to the required amount of acceptor particle dispersion (resulting in a total volume of approximately 1.5 mL in each transfer vial). For each time point of sampling, a separate transfer vial was used. During transfer, the samples were placed on a horizontal shaker (Vibrax VXR Basic, IKA-Werke GmbH & Co. KG, Staufen, Germany) and agitated with 300 rpm at ~23 °C. Samples were withdrawn using a 2 mL plastic syringe via filtration at predetermined time points. For this purpose, a polyethersulfone (PES) membrane with 1.2 μm pore size (Pieper Filter GmbH, Bad Zwischenahn, Germany) was mounted into a custom-built screw cap that was attached to the transfer vial shortly before sampling.

For fenofibrate and retinyl acetate, the drug load of the donor nanoemulsions, as well as the remaining amount of drug in the nanoemulsions during transfer experiments, was quantified via UV spectroscopy (Specord 40, Analytik Jena AG, Jena, Germany). Samples were dissolved in tetrahydrofuran/water 9/1 (*v/v*) and measured at wavelengths of 287 nm (fenofibrate) or 360 nm (retinyl acetate) three times. Where required, the measured absorptions were corrected for the blank absorptions of the dissolved unloaded nanoemulsion that had been treated in the same way as the respective drug-containing nanoemulsion. Calibration curves for each drug were obtained by preparing at least six different dilutions containing varying amounts of the respective drug. The amount of transferred drug was calculated by subtracting the amount in the sampled aqueous donor system from the originally applied one.

Orlistat could not be quantified via UV spectroscopy in the presence of trimyristin because of overlapping absorption signals. Thus, orlistat transfer from the trimyristin nanoemulsion into the lipophilic acceptor was investigated by DSC. The change in crystallization temperature (ΔT_cryst._, determined upon cooling) is in a linear relation to the decrease in drug content [22]. The respective donor emulsion, diluted with water in the same volume as that of the acceptor system (which corresponded to 0% drug transfer), and an unloaded trimyristin nanoemulsion with comparable characteristics (corresponding to 100% drug transfer; measured as control) were used to calculate the transferred amount of orlistat by applying the rule of three. Control experiments were performed by incubating unloaded trimyristin emulsion with the lipid-containing microsphere dispersion under the same conditions. Fluctuations in the crystallization temperature of the donor emulsion that were not caused by drug transfer could thus be identified and included into the calculations of the transferred amount of drug [22].

#### 3.9.2. Albumin Solution as Acceptor

The overall amino acid sequence identity of BSA in comparison to human serum albumin (HSA) is ~76%, leading to a similar tertiary structure of these molecules, with similar binding sites [40,41]. In order to evaluate the contribution of albumin as a potential acceptor for lipophilic drugs in blood, BSA was used for screening purposes in this study.

BSA solution was freshly prepared before use by dissolving 43 mg/mL BSA in PBS buffer under continuous stirring at 200 rpm. This concentration was chosen for two reasons. First, the concentration of albumin in human plasma is 35–50 mg/mL [23] and, second, the concentration of lipid acceptor in the studies performed here with CNTM-containing dispersion that was incorporated in microspheres was in the same range (cf. Section 2.1.3). Thus, the chosen BSA concentration offered the possibility to compare the contribution of (different) available acceptor structures used in this study on the one hand, and also provided a realistic approach to the albumin concentration in vivo.

A total of 70 µL of the drug-loaded donor emulsions were mixed in a ratio of 1 + 9 (ratio adjusted based on the lipid quantification in the donor emulsion, as determined using DSC) with the acceptor solution in a 3 mL vial and incubated for 24 h in the same way as described above (total volume ~1.5 mL/vial). At various time points, samples were withdrawn using an Eppendorf pipette and directly measured by DSC. In contrast to the hydrogel-bead-based setup, no filtration step was necessary using this method. The change in crystallization temperature (ΔT_cryst._) determined upon cooling was used to calculate the transferred amount of each drug under investigation, as described in Section 3.9.1 for orlistat. For the control experiments, an unloaded trimyristin emulsion with comparable characteristics was incubated in the acceptor solution and in PBS buffer, and measured by DSC as well.

All transfer results are presented by plotting the fraction of transferred drug (%) against the time (hours, h). All transfer studies were performed in triplicate.

## 4. Conclusions

The liquid (trimyristin) and liquid crystalline (cholesteryl nonanoate) state, as well as the integrity of the nanoparticles, could be preserved during hydrogel bead production, and the resulting system was successfully applied as lipophilic acceptor in transfer studies. The course of transfer observed using the lipid-containing hydrogel particles as the acceptor was in relation to the lipophilicity of the drugs: the higher the logP value, the slower the transfer. In all cases, the partition equilibrium of the drugs under investigation was found to be in favor of the trimyristin emulsion droplets. Given that there is no officially approved method to investigate the release of lipophilic drugs from nanosized carriers, the hydrogel-bead-based setup can be helpful in order to compare the contribution of different lipophilic acceptors to the release performance of colloidal drug delivery systems. The nature of the lipophilic acceptor in release studies is essential, as it strongly affects the release behavior. No detectable fraction of the drugs was transferred to BSA, demonstrating clearly that albumin seemed to be of minor importance as lipophilic acceptor for the drugs under investigation in the present study. Albumin as solubilizing agent to be used in transfer experiments should thus be evaluated thoughtfully, especially for drugs with high lipophilicities. The lipophilic substances used in the present study as an acceptor were selected as model compounds in order to mimic different lipophilic acceptors present in the blood. Thus, a closer approach to the physiological environment was provided than with many other release media currently applied. However, many other aspects, e.g., the physical state of the acceptor particles and, with special regard to the intravenous route of administration, a realistic dilution of the donor system, should be taken into consideration for future investigations, as they may also affect the drug release performance.

## Figures and Tables

**Table 1:** Particle sizes, lipid content, and drug load of the different nanodispersions used as donors in transfer studies or as acceptor particles to be incorporated into the hydrogel microbeads.

Nanodispersion	Z-Average (nm)	PdI	Lipid Content (%) DSC	Lipid Content (%) HPLC	Drug Load Related to Trimyristin (%)
**Trimyristin donor emulsions**					
FFB donor	126	0.09	9.66	-	2.89
RA donor	113	0.10	9.97	-	2.94
ORL donor	115	0.09	9.36	-	3
**Acceptor dispersions** **(*n* = 3 batches ± SD)**					
Trimyristin (TM)	118 ± 4	<0.10	9.60 ± 1.01	9.53 ± 1.07	-
Cholesteryl nonanoate (CN)	133 ± 0.8	<0.11	-	9.08 ± 0.16	-
Mixed trimyristin– cholesteryl nonanoate (CNTM)	132 ± 0.2	<0.10	8.91 ± 0.30	9.03 ± 0.16	-

## Data Availability

Data is contained within the article and supplementary material.

## Supplementary Materials

The following are available online at https://www.mdpi.com/article/10.3390/ph14090865/s1, Figure S1: Lipid determination of an exemplary batch of different lipid nanodispersions used as acceptor particles to be incorporated into the hydrogel beads.
