# Supplementary material for: Transfer Investigations of Lipophilic Drugs from Lipid Nanoemulsions to Lipophilic Acceptors: Contributing Effects of Cholesteryl Esters and Albumin as Acceptor Structures
Source: Pharmaceuticals (Basel). 2021 Aug 28;14(9):865. doi: 10.3390/ph14090865 (PMC8471859; doi:10.3390/ph14090865)
Supplement: Supplementary file 1 [file pharmaceuticals-14-00865-s001.zip › pharmaceuticals-1341373-supplementary.pdf]

Supplementary Material

for

Transfer investigations of lipophilic drugs from lipid nanoemulsions to lipophilic acceptors: Contributing effects of cholesteryl esters and albumin as acceptor structures

Sabrina Knoke[1], Heike Bunjes[1,2*]

[1] Technische Universität Braunschweig, Institut für Pharmazeutische Technologie und Biopharmazie, Mendelssohnstraße 1, 38106 Braunschweig, Germany

[2] Zentrum für Pharmaverfahrenstechnik, Franz-Liszt-Straße 35a, 38106 Braunschweig, Germany

* Corresponding author, Tel: +49 531 391 5652; E-Mail: heike.bunjes@tu-braunschweig.de

Correlation of lipid quantification as performed using DSC and HPLC

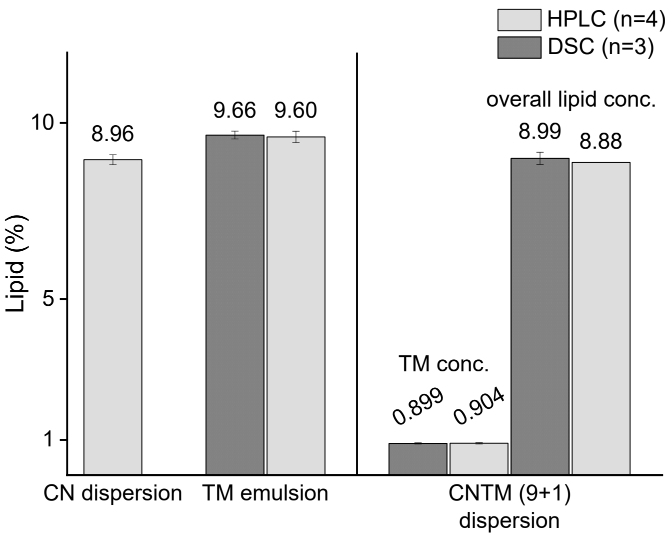

Figure S1. Lipid determination of an exemplary batch of different lipid nanodispersions used as acceptor particles to be incorporated into the hydrogel beads. DSC (patterned bars; n= 3 measurements ± SD) and HPLC (uniform bars; n= 4 measurements ± SD) results. The CNTM dispersion was prepared by mixing the pure dispersions of cholesteryl nonanoate (CN) and trimyristin (TM) in a ratio of 9 + 1. The overall lipid concentration (overall lipid conc.) of the CNTM dispersion was calculated by multiplying the trimyristin concentration (TM conc.) as determined via DSC by ten. The respective overall lipid concentration determined by HPLC is the sum of the results of the pure dispersions determined individually. Comparing these two methods, the lipid determination revealed very similar results in both cases. Thus, applying the DSC method for the determination of the overall lipid content in the hydrogel beads seemed appropriate. The slight reduction as compared to the weighed-in lipid concentration is most likely related to dilution with process water that remained in the homogenization device during manufacturing.
